# Photocatalytic Oxidation of HMF under Solar Irradiation: Coupling of Microemulsion and Lyophilization to Obtain Innovative TiO_2_-Based Materials

**DOI:** 10.3390/molecules25225225

**Published:** 2020-11-10

**Authors:** Alessandro Allegri, Valeriia Maslova, Magda Blosi, Anna Luisa Costa, Simona Ortelli, Francesco Basile, Stefania Albonetti

**Affiliations:** 1 Dip. Chimica Industriale “Toso Montanari”, Università di Bologna, Viale Risorgimento 4, 40136 Bologna (BO), Italy; alessandro.allegri2@unibo.it (A.A.); f.basile@unibo.it (F.B.); 2 ISTEC-CNR, Institute of Science and Technology for Ceramics, National Research Council, Via Granarolo 64, 48018 Faenza, Italy; magda.blosi@istec.cnr.it (M.B.); anna.costa@istec.cnr.it (A.L.C.); simona.ortelli@istec.cnr.it (S.O.)

**Keywords:** 5-hydroxymethyl furfural, spray-freeze drying, photocatalysis, TiO_2_, microemulsion

## Abstract

The photocatalytic oxidation of biomass-derived building blocks such as 5-hydroxymethylfurfural (HMF) is a promising reaction for obtaining valuable chemicals and the efficient long-term storage of solar radiation. In this work, we developed innovative TiO_2_-based materials capable of base-free HMF photo-oxidation in water using simulated solar irradiation. The materials were prepared by combining microemulsion and spray-freeze drying (SFD), resulting in highly porous systems with a large surface area. The effect of titania/silica composition and the presence of gold-copper alloy nanoparticles on the properties of materials as well as photocatalytic performance were evaluated. Among the lab-synthesized photocatalysts, Ti_15_Si_85_ SFD and Au_3_Cu_1_/Ti_15_Si_85_ SFD achieved the higher conversions, while the best selectivity was observed for Au_3_Cu_1_/Ti_15_Si_85_ SFD. The tests with radical scavengers for both TiO_2_-m and Au_3_Cu_1_/Ti_15_Si_85_ SFD suggested that primary species responsible for the selective photo-oxidation of HMF are photo-generated electrons and/or superoxide radicals.

## 1. Introduction

Among the bio-based molecules, 5-hydroxymethyl furfural (HMF) is one of the main candidates in replacing the oil-derived platform molecules in the production of polymers, chemicals, and fuels. The global market of HMF is fast-growing, and in 2022 it is estimated to be 123 billion USD [1]. The number of scientific works, reporting on the use of HMF as a starting material, has been increasing annually to a wide extent. By means of selective oxidation, HMF can be converted to 2,5-furandicarboxylic acid (FDCA). Together with HMF, FDCA is known as a “sleeping giant” because of its enormous market potential [2,3]. For instance, FDCA can be used as an oil-free alternative to terephthalic acid in polymer manufacturing [4]. Moreover, HMF can be also selectively oxidized to 2,5-diformylfuran (DFF), a precursor for pharmaceutical and antifungal agents, furanic biopolymers, and furan-urea resins [5]. The molecule of 2,5-dimethylfuran (DMF) can be obtained from HMF hydrogenation reaction. DMF is considered as a potential biofuel [6]. Meanwhile, the reduction of HMF gives 2,5-bis(hydroxymethyl) furan (BHMF), known as an intermediate in the pharmaceutical industry, and the synthesis of crown ethers and polymers [3]. In specific conditions, HMF can undergo a ring-opening reaction, where the main products are levulinic and formic acids [7]. Levulinic acid is a building block in polymer industry and production of bio-solvents. Meanwhile, hexanediol is extensively used in polymer production, coatings, and adhesives [8].

In particular, poly(ethylene furanoate), PEF, can be successfully produced by polycondensation of FDCA with ethylene glycol. Recent studies have demonstrated that PEF is a 100% renewable polymer with an enhanced oxygen and carbon dioxide permeability compared to poly(ethylene terephthalate), PET, despite a greater CO_2_ solubility observed for PEF [8]. The industrial commercialization of PEF material is expected by 2023 [9]. Among examples, Dupont and Avantium are leaders in pilot and flagship plant development for PEF production. Recently, Motagamwala and co-workers [10] have reported that Avantium has developed an FDCA production process in which methoxymethylfurfural is used as the substrate, while Co(OAc)_2_, Mn(OAc)_2_, and HBr are the homogeneous catalytic systems in acetic acid medium. Such approach leads to 96% of FDCA, avoiding the problems associated with the low stability of HMF in acidic/basic conditions, low solubility of FDCA in common solvents, and incomplete oxidation of HMF to FDCA. However, the remained insoluble fractions of intermediates of oxidation require their further purification. Moreover, the use of corrosive media and dangerous compounds triggers some questions regarding the sustainability of the process.

**Scheme 1:**
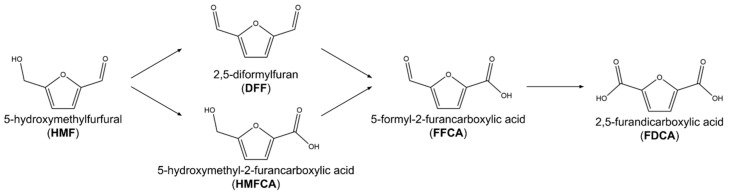
Reaction scheme of HMF partial oxidation.

Therefore, the preparation of active and stable metal-supported catalytic systems able to perform HMF oxidation in aqueous media are currently receiving a great interest [11]. The accepted pathway of HMF oxidation to FDCA is depicted on Scheme 1, showing the intermediates products.

Numerous studies have been performed to date on the heterogeneous selective oxidation of HMF to FDCA. Mainly Au, Pd, Pt, and also these metals in the form of alloy supported on metal oxides (TiO_2_ [4,7,12,13], CeO_2_ [4,14], NiO [15], etc.) have shown an improved activity towards FDCA operating at mild temperatures (50–90 °C), in the presence or absence of base as co-catalyst. Davis and co-workers [16] emphasized the pivotal role of inorganic base in fast transformation of HMF to HMFCA. However, the base also leads to a formation of insoluble oligomers (humins), which lower the overall atom efficiency of the process. On the other hand, higher temperatures have to be applied to make HMF to FDCA oxidation process feasible in the base-free conditions [17].

In the last decade, selective conversion of HMF using photoactive materials have shown a growing interest since it implies the use of solar light as energy source, ambient temperature and pressure, and ideally water as a green solvent. Among the semiconductors tested in photocatalytic conversion of HMF up to date, the materials based on TiO_2_, g-C_3_N_4_, Nb_2_O_5_, MnO_2_, rGO, ZnO, tetraalkylammonium decatungstates, and Bi_2_MoO_6_ were reported [18,19,20,21,22,23].

The choice of solvent has a substantial effect on a photo-catalytic performance. For instance, ascribing to a better oxygen dissolution in benzotrifluoride, the yield of DFF reached as high as 90% at 19% of HMF conversion in this solvent, using Nb_2_O_5_ under visible light [24]. In other organic solvent such as toluene, the photoactivity of rGO-supported Au-Ru nanoparticles reached 95% of HMF conversion with 95% and 5% of DFF and FFCA selectivities, respectively [25]. The effect of solvent was also shown in the work reporting the performance of manganese (IV) oxide nanorods [26]. The authors showed a negligible reactivity of MnO_2_ nanorods in water and in the mixture with acetonitrile (50:50), explained by a lower solubility and poor accessibility of oxygen to reach the surface of photoactive material due to the hindrance of its active sites by water molecules. On the other hand, the reactivity of MnO_2_ nanorods in acetonitrile was substantially greater compared to aqueous media even in the dark, showing 63% of HMF conversion with 92% selectivity of DFF, while the test under visible light resulted in 83% of HMF conversion and 93% selectivity of DFF. Nevertheless, the best result was achieved using LED as a source of light (375 nm, 4 h, and 39 °C), yielding 100% of DFF at 99% of HMF conversion. The activity of three types of tetraalkylammonium decatungstates and the effect of various additives on HMF photo-oxidation in acetonitrile as a reaction medium were also reported [19]. Among the alkyl moieties, tetramethylammonium decatungstate showed a better conversion of HMF (59%) compared to tetrapropylammonium and tetrabuthylammonium decatungstates with 53% and 49% of HMF conversion, respectively. These results stem from the improved synthetic quality, stability and redox capacity of a shorter alkyl chain. In the same work, HBr as an additive showed improved photo-catalytic oxidation of HMF, carbon balance and DFF yield over all the tested range of HMF concentrations (0.02–0.08M) compared to other additives (H_2_O, DMSO, NaBr, [Bimi]Cl, HCl, HI, H_2_SO_4_, HAc), reaching as high as 92% of HMF conversion at 72% of carbon balance. This is due to peculiar role of HBr as stabilizer of decatungstate structure and excited state under light irradiation, and as a restraint for HMF from polymerization and, thus, by-production formation.

Several recent studies on photocatalytic selective HMF oxidation have been carried out by using composites based on graphitic carbon nitride (g-C_3_N_4_) [22,27,28,29,30,31,32,33]. The bandgap of this material is 2.7 eV, which enable g-C_3_N_4_ to absorb in the visible range of solar spectrum, where the light is generated with the highest irradiance. Additionally, the energy of valence band of g-C_3_N_4_ is 1.4 eV, that makes difficult the formation of hydroxyl radical (^•^OH), highly oxidizing species. The key role in photo-oxidation of HMF by g-C_3_N_4_-based catalysts authors ascribed to superoxide radical (^•^O_2_^−^), acting as a milder oxidizing species, thus, exhibiting outstanding results in aqueous environment. To specify, I. Krivtsov and co-workers [27] obtained 50% selectivity of DFF at 40% of HMF conversion using exfoliated carbon nitride in aqueous media under natural solar light. Furthermore, g-C_3_N_4_-based materials were successfully tested in a pilot plant in Plataforma Solar de Almería (Spain) by the group of M. Ilkaeva [29]. Recently, the group of H. Zhang [32] have demonstrated selective conversion of HMF reaching 27% with WO_3_/g-C_3_N_4_ composite under visible light, and ascribed the high DFF selectivity (87%) to a better electron-hole pair separation in the composite. Secondly, authors suggested both ^•^O_2_^−^ and h^+^ are responsible for HMF photo-oxidation based on tests with scavengers. Alternatively, Y. Zhu and co-workers [22] reported visible-light-induced photocatalytic performance in aqueous media using g-C_3_N_4_/NaNbO_3_ composite. The authors achieved 36% of HMF conversion and 87% of FFCA selectivity after 8 h of reaction with the optimal amount of NaNbO_3_ and Na_2_CO_3_ as co-catalyst. With a longer time, the authors observed further oxidation of FFCA into FDCA. In the work of Elisa I. García-López et al. [23], the impregnated porphyrin groups with and without metal complexes improved visible-light absorption of graphitic or thermo-exfoliated carbon nitride. These materials were tested under natural solar light in aqueous solution of HMF. The best result was achieved at pH 9 for Cu-containing porphyrin complex loaded on thermo-exfoliated carbon nitride, giving 38% of DFF selectivity at 72% of HMF conversion.

Another approach was proposed by Gonzalez-Casamachin et al. [21]. In their work, ZnO nanoparticles were incorporated into conducting polymer, polypyrrole. The results obtained with this material showed formation of HMFCA, FFCA, DFF, and FDCA with selectivities of 55%, 12.4%, 0.4%, and 30%, respectively, at 25 °C and around 5% of HMF conversion under visible light in water. The conversion was substantially improved at higher temperature (35 and 40 °C), though compromising the selectivity of valuable products. Alternatively, active under visible light Bi_2_MoO_6_-supported 1.5% Fe (III) clusters showed 95% of DFF selectivity with 33% of HMF conversion in water [34].

To date, titanium dioxide remains in the front line as the most cited material in photocatalytic community, because of its photo-stability, non-toxicity, and easiness of preparation. The first work on photocatalytic oxidation of HMF in water using TiO_2_ was published by Augugliaro et al. [35], which reported the use of TiO_2_ nanoparticles in different polymorphs. The results showed that high specific surface area, low crystallinity, and reduced hydroxylation degree of synthesized TiO_2_ are among the influencing parameters, showing a less significant effect on the over-oxidation of HMF to CO_2_ and H_2_O through the formation of DFF and some aliphatic intermediates. This has also been confirmed in other studies using differently synthesized titania [20,36,37]. A tendency to mineralization is an expected feature since, under UV light, TiO_2_ in water media is known to generate ^•^OH, ^•^O_2_^−^, and HO^•^_2_ radicals, leading to a complete oxidation of aliphatic and aromatic compounds [38,39]. A different behavior was reported for Au-decorated TiO_2_ (P25) [40], giving almost complete conversion of HMF to HMFCA in basic conditions, where the role of Na_2_CO_3_ was suggested as ^•^OH scavenger.

Exploiting better the beneficial effect of large surface area on HMF photo-oxidation [23,35,37], the present work describes the activity of novel titania-based materials prepared in a three-steps approach. Firstly, (i) nanocrystalline titania was prepared via microemulsion method, the activity of which was compared with commercial TiO_2_ samples, DT51 and P25. Following the next step, (ii) lab-synthesized titania was matrix encapsulated with a commercial silica, and, lastly, (iii) subjected to the spray-freeze drying (SFD) step, resulting in a high surface area and low bulk density of the material. SFD is a method, in which thermo-labile compounds can be dried at low temperatures, producing high-surface-area spherical porous systems [41]. For catalyst preparation, freeze-drying has been suggested to reduce precursor solution mobility upon drying and, therefore, to control the location of deposition of the precursor phase. Moreover, in this work, a visible-light absorption through the surface plasmon resonance (SPR) phenomenon was enhanced and realized by introducing pre-formed gold-copper alloy at a SFD step, since this technique allows a homogeneous embedding of the active phases into the support, minimizing the possibility of phase separation on a molecular scale, as also demonstrated for drugs. Nevertheless, few applications have been reported in the catalytic field until now [41,42,43].

## 2. Results

The present work describes the activity of titania-based materials prepared using nanocrystalline lab-made titania synthesized via microemulsion method in the photo-oxidation of HMF under solar simulated conditions. The silica-containing mixed oxides were produced at different compositions using a colloidal heterocoagulation method associated with the spray-freeze drying [41]. Similar procedure was used to prepare gold-copper containing photocatalysts (metal total loading 1.5 wt%). Table 1 reports the composition and main characteristic of the materials discussed in this work.

### 2.1. Catalyst Characterization

#### 2.1.1. Characterization of Commercial and Microemulsion-Synthesized TiO_2_

Three different types of TiO_2_ samples were characterized in terms of their crystalline phase and size, specific surface area, absorption of light and bulk density. Table 1 shows that lab-synthesized TiO_2_-m prepared via microemulsion method has a greater specific surface area lower crystalline size than commercial samples. This is characteristic for microemulsion synthesis to deliver small crystallites with high surface area [44,45,46,47]. Regarding the crystalline phase, TiO_2_-m is mainly represented by anatase polymorph with rutile and brookite as minor phases. Commercial P25 and DT51 catalysts are composed by anatase-rutile mixed phase, and pure anatase, respectively (Table 1). DRS analysis was used to determine the energy bandgap of the materials. In Table 1, it can be seen that TiO_2_-m has a smaller bandgap energy, thus its edge of absorption is more a red shifted, followed by P25 and DT51. The shift of absorption could be provoked by the presence of formed defects upon the synthesis or calcination, among which are oxygen vacancies and Ti^3+^ ions known to enhance the visible-light response of semiconductors [48,49]. Nevertheless, all the samples are absorbing in the UV part of solar spectrum. It is also known that the morphology of powder, namely its porosity, aggregation of particles, texture, and bulk density, influences the photocatalytic activity [50,51]. Therefore, SEM characterization of these powders (Supplementary Figure S1) has to be considered. The commercial DT51 has homogenously distributed small, round-shape particles, while the particles of P25 has a very porous, sponge-like form. The synthesized TiO_2_-m consists of small, but highly aggregated particles. Using SFD process for TiO_2_-m SFD production prevents powder sintering, leading to material with a high surface area.

Bulk densities were also taken into account. Table 1 shows that P25 and TiO_2_-m SFD have the lower values, followed by DT51 and TiO_2_-m. A low bulk density may indicate the presence of a more exposed surface provided by the pores with intrinsic connectivity which, in theory, allows for efficacious charge transfer and reactants mass flow.

#### 2.1.2. Characterization of SiO_2_-TiO_2_-m Mixed Oxides

**Figure 1:**
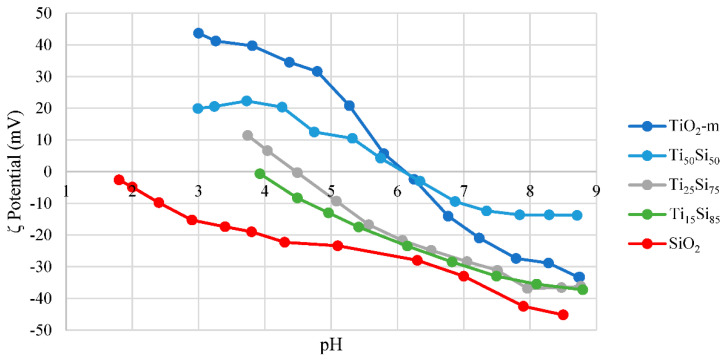
ζ-potential titration of titania-silica suspensions before spray-freeze drying.

The SiO_2_-TiO_2_-m mixed oxides were prepared at different compositions (TiO_2_-m to SiO_2_ as 50:50, 25:75, and 15:85 as weight %) using a colloidal heterocoagulation method, which combines positively charged TiO_2_ and negatively charged SiO_2_ nanoparticles, coupled by means of the spray-freeze drying process. Sols containing the different oxides precursors were analyzed by ζ-potential and the corresponding spray-freeze dried granules were morphologically investigated by FESEM. Figure 1 shows ζ-potential titrations in a pH range from 0 to 9, indicating the dependence of the colloidal behavior with the composition. The graph highlights that by increasing the SiO_2_ amount, the ζ-potential/pH curves of mixed oxides move toward the SiO_2_ curve, characterized by negative ζ-potential values along almost all the pH range. This behavior could be explained by a “matrix encapsulation” [52] upon the formation of SiO_2_-TiO_2_-m composite’s suspension, when TiO_2_-m particles are trapped inside the silica structure.

**Figure 2:**
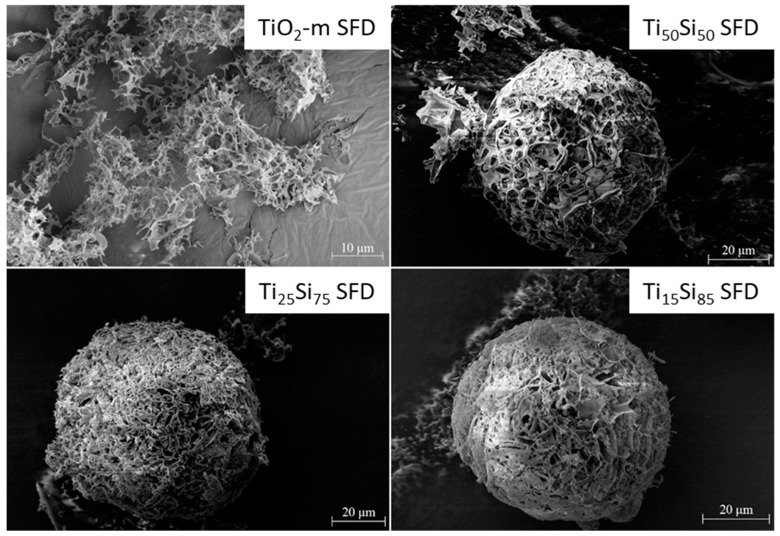
SEM images of the spray-freeze dried powders.

**Figure 3:**
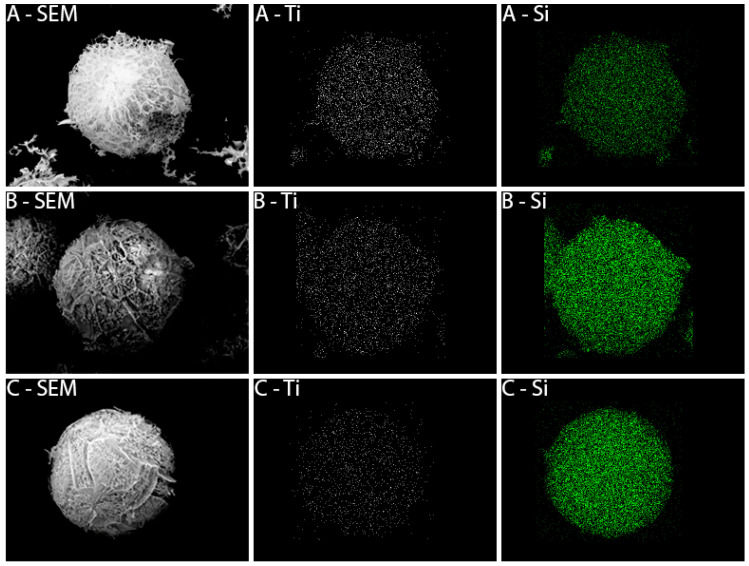
SEM-EDX maps of the samples Ti_50_Si_50_ SFD (**A**), Ti_25_Si_75_ SFD (**B**) and Ti_15_Si_85_ SFD (**C**). From left to right, SEM images, Ti maps (white) and Si maps (green).

Figure 2 shows that the incorporation of silica in spray-freeze dried samples substantially improves the structure of granules, making them spherical and uniform, which is in a good accordance with previously reported works [37]. Moreover, Figure 3 shows SEM-EDX maps, displaying a homogeneous distribution of the phases without any apparent segregation. This is also confirmed by the measurements of bulk density of the SFD composites (Table 1), demonstrating a substantial reduction in bulk density (and more evidently with the increase of silica content, TiO_2_-m SFD > Ti_50_Si_50_ SFD > Ti_25_Si_75_ SFD > Ti_15_Si_85_ SFD) compared to initial TiO_2_-m and commercial samples (Supplementary Figure S1).

#### 2.1.3. Characterization of Ti_15_Si_85_ SFD and TiO_2_-m SFD Supported Au_3_Cu_1_ Nanoparticles

**Figure 4:**
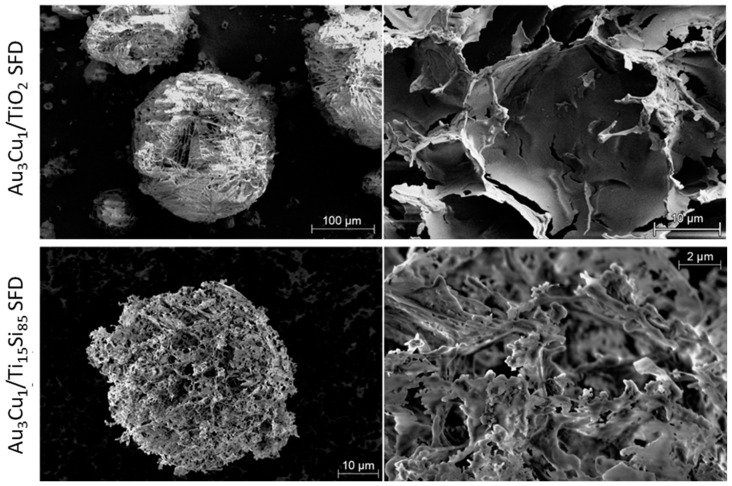
SEM images of the Au_3_Cu_1_—containing spray-freeze dried powders.

Using the same SFD procedure, gold-copper containing photocatalysts were prepared adding pre-formed bimetallic nanoparticles (Au_3_Cu_1_) to the water suspension of the oxides. Figure 4 shows SEM images of Au_3_Cu_1_-containing samples. Both samples retained a highly porous homogeneous morphological structure of granules as an undecorated Ti_15_Si_85_ SFD sample, indicating that the introduction of nanoparticles does not change the spray-freeze drying process. Moreover, the shape of Au_3_Cu_1_/TiO_2_-m SFD was substantially improved compared to pristine TiO_2_-m SFD with a more irregular form (Figure 2). This can be possibly explained by the presence of polymeric agent in sols of nanoparticles, stabilizing the granules upon the SFD step.

**Figure 5:**
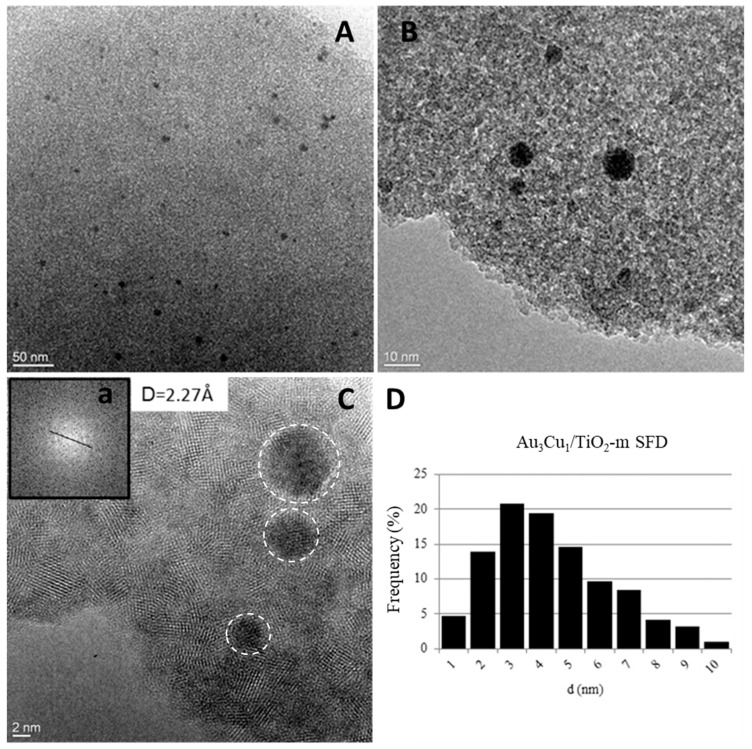
HR-TEM images (**A**–**C**) and histogram (**D**) of particle’s dimension of Au_3_Cu_1_ nanoparticles deposited on TiO_2_-m SFD. The inset (**a**) in figure C shows the FFT of a representative nanoparticle (distance at 2.27 Å).

Figure 5 shows TEM images of the Au_3_Cu_1_ nanoparticles deposited on TiO_2_-m SFD, demonstrating the presence of spherical and well-dispersed metal particles with a narrow particle size distribution centered on 4.6 nm, (Figure 5D). The absence of large agglomerates of Au_3_Cu_1_ nanoparticles, thanks to the use of SFD process, can be observed from TEM images at high and low magnifications. The images highlight the presence of titania nanoparticles as well, which maintained a small diameter in the 6-8 nm range despite they underwent the SFD treatment (Figure 5C). HAADF-STEM analysis and EDX mapping (Supplementary Figure S2) of nanoparticles revealed an amount of Cu content in Au nanoparticles similar to the theoretical ones. Moreover, HR-TEM images revealed that particles exhibited single crystalline structures with lattice fringes giving main d-spacing of 0.227 nm (Figure 5C-a, marked section). It should be noted that the interplanar spacing of the (111) plane in pure Au and Cu crystal are 2.355 Å and 2.087 Å, respectively [53]. The observed single interplanar spacing of 2.27 Å therefore indicates the formation of fcc solid solution between Au and Cu atoms in the nanoparticle.

For the samples Au_3_Cu_1_/TiO_2_-m SFD and Au_3_Cu_1_/Ti_15_Si_85_ SFD the measurements to determine the bulk density and specific surface area were carried out as well and presented in Table 1. The values of bulk density for Au_3_Cu_1_-decorated titania are in good agreement with those of pristine supports, suggesting that deposition of alloy nanoparticles did not change the porosity and morphological structure of powders. The deposition of nanoparticles also did not show a substantial effect on specific surface area.

### 2.2. Photocatalytic Tests

#### 2.2.1. HMF Photo-Oxidation Reactions

**Figure 6:**
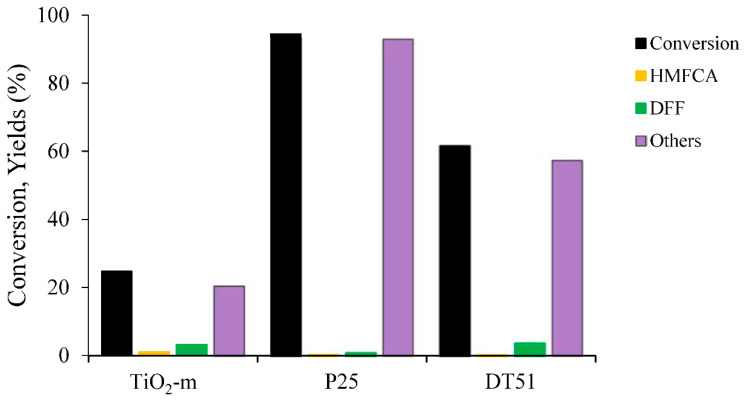
Photocatalytic activity of different titania powders. Reaction conditions: t = 1 h, T = 30 °C, P = 1 atm O_2_, m_cat_ = 20 mg, V = 20mL, [HMF]_0_ = 5 × 10^−4^ mol/L.

Photocatalytic HMF oxidation tests were carried out on commercial and microemulsion-synthesized TiO_2_ catalysts using simulated solar light in the base-free conditions. Within bare supports (Figure 6), P25 and DT51 commercial catalysts are very photo-active, converting 94% and 62% of HMF by P25 and DT51, respectively, mainly to CO_2_ and other by-products. Traces of HMFCA and DFF were observed with selectivity of HMFCA below 1% for both samples, and selectivity of DFF of 1% and 6% for P25 and DT51, respectively. On the other hand, TiO_2_-m demonstrates milder oxidation, showing 25% of HMF conversion with improved selectivity to DFF and HMFCA, resulted in 13% and 4 %, respectively. A more selective photo-oxidation by TiO_2_-m could be explained by two reasons. Firstly, as was supported by the results of DRS measurements, TiO_2_-m may develop a presence of some defective sites (oxygen vacancies, uncoordinated Ti ions, grain boundaries, etc.) [54,55], which suppress the generation of reactive oxygen species. Secondly, a larger bulk density compared to commercial analogues may limit the conversion of HMF due to the restricted availability of exposed surface for the reagent by the highly agglomerated particles of TiO_2_-m.

**Figure 7:**
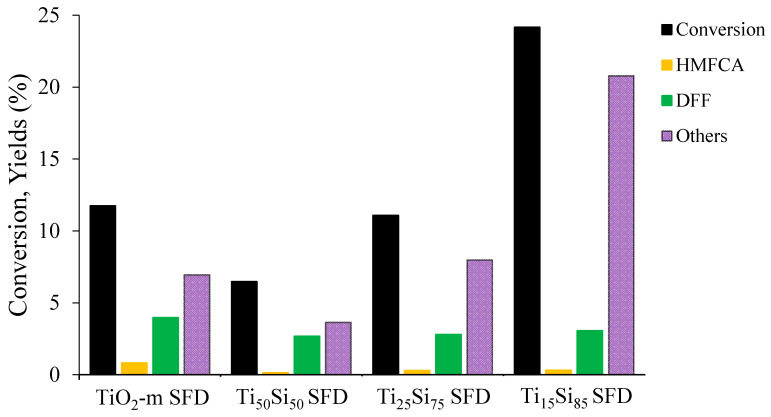
Photocatalytic performances of titania-silica spray-freeze-dried nanocomposites at different compositions. Reaction conditions: t = 1 h, T = 30 °C, P = 1 atm O_2_, m_cat_ = 20 mg, V = 20 mL, [HMF]_0_ = 5 × 10^−4^ mol/L.

With the aim to reduce the detrimental effect of synthesized TiO_2_-m aggregation phenomenon on photocatalytic activity in aqueous environment, TiO_2_-m paste after the synthesis, just before the drying step, was subjected to SFD process. Figure 7 demonstrates the activity of SFD catalysts in different titania-silica weight ratio. Despite a more porous morphological structure of TiO_2_-m SFD compared to initial TiO_2_-m, the conversion of HMF decreased to 12%. However, the selectivity of HMFCA and DFF was notably improved, amounting to 7% and 34%, correspondingly. With the increase of silica fraction to 50% in titania-silica composites, the HMF conversion decreased. The further increase of silica content caused an increase of HMF conversion, but the product yield was not proportionally affected. With the 85% of silica content, HMF conversion achieved 24%, though compromising carbon balance of reaction. The results point out an overall positive synergistic interaction between silica and titania. The silicon oxide is known to be photocatalytically inert, but its presence promotes a significant improvement of the TiO_2_ conversion. This effect is quite evident normalizing HMF conversion versus TiO_2_ content in the specific sample (Figure S3). We hypothesized that the SiO_2_ action is due to the dispersion capability of the silica, consistent with the surface area increase, as also demonstrate in our previous work [41]. Silica could decrease the H_2_O_2_ formation at the particles interfaces, as described in the literature [56], possibly through the inhibition of the parallel reactions of oxygen reduction and water oxidation, thus increasing the intermediates radicals, ^•^OH, and boosting the reaction.

To elucidate the role of plasmonic nanoparticles on photo-oxidation of HMF, and to lower the cost of prepared catalyst, gold-copper alloy nanoparticles were deposited on TiO_2_-m SFD and Ti_15_Si_85_ SFD supports. The choice of Au-Cu nanoparticles deposited on titania was also driven by the improved results of catalytic partial oxidation of HMF towards the furan dicarboxylic acid (FDCA) production as compared to monometallic gold due to a better sample stability and resistance to poisoning [4,7]. With the aim to find an optimal gold–copper ratio, the preliminary screening tests were carried out. Supplementary Figure S4 shows that 3:1 is the optimal molar ratio of Au and Cu, resulting in a greater conversion of HMF and yields of products compared to other ratios, as was evidenced in other photo-catalytic reactions [57,58].

**Figure 8:**
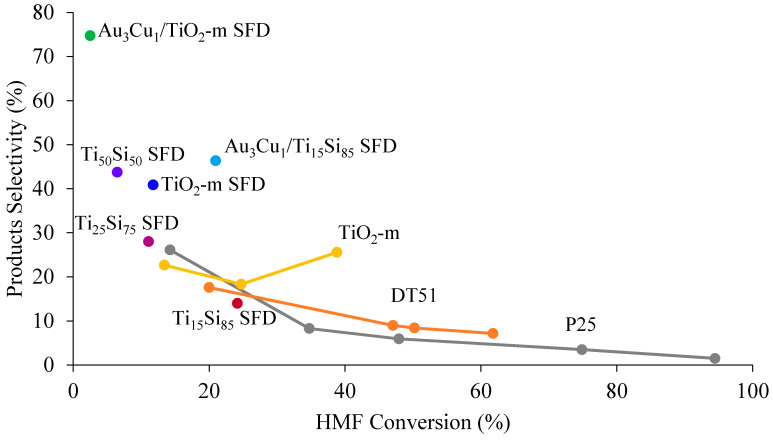
Effect of conversion on the total products selectivity of different titania-based photocatalysts. Reaction conditions: T = 30 °C, P = 1 atm O_2_, m_cat_ = 20 mg, V = 20 mL, [HMF]_0_ = 5 × 10^−4^ mol/L.

Figure 8 shows the photo-catalytic activity of all studied samples reported as a conversion selectivity plot to reveal similarity and differences between samples gold-copper. The pristine materials (P25, DT51 and TiO_2_-m) firstly showed similar trend at the early stage of reaction, up to 25% of HMF conversion. Then, the commercial samples (P25 and DT51) showed a gradual decrease in the selectivity of products with the increase in HMF conversion. In contrast to commercial samples, TiO_2_-m showed better selectivity at a higher conversion of HMF.

The test with Au_3_Cu_1_/Ti_15_Si_85_ SFD sample having 85% of silica content resulted in an increase of HMF conversion from 3% to 21% as compared to the sample without silica. This suggests that despite of a lower surface area of the Au_3_Cu_1_/Ti_15_Si_85_ SFD sample, the presence of silica in the system and a lower bulk density associated with a more homogeneous porous morphology of the powder, favorably affects the conversion of HMF. The comparison of all the catalysts tested, reported in Figure 8, revealed that in general microemulsion-prepared systems showed greater selectivity than commercial samples. Noteworthy, in the presence of Au_3_Cu_1_ nanoparticles the yield and selectivity of desirable products substantially improved (Table 2) in comparison to supports alone, indicating the different mechanism of reaction undergone in the presence of plasmonic particles.

#### 2.2.2. Study of the Mechanism by Tests with Radical Scavengers

**Scheme 2:**
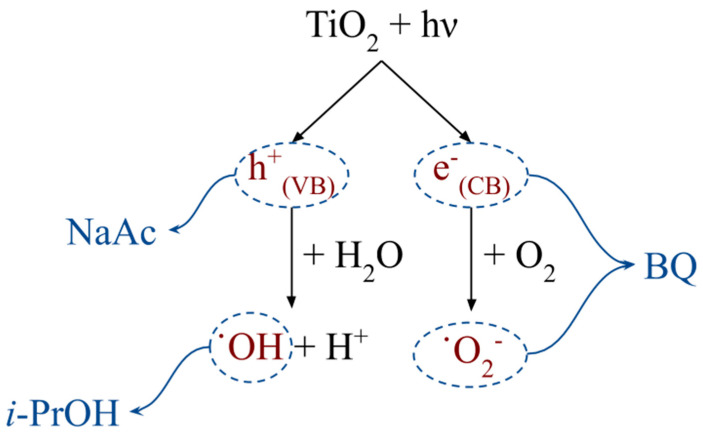
Process of photo-activation of titania in water. The species responsible for the conversion of HMF are marked in red, while the scavengers used are marked in blue. NaAc: Sodium Acetate; i-PrOH: 2-Propanol; BQ: BenzoQuinone.

To investigate deeper the differences in the two mechanisms undergone in the presence and absence of Au_3_Cu_1_ nanoparticles, the tests with radical scavengers were carried out for TiO_2_-m and Au_3_Cu_1_/Ti_15_Si_85_ SFD. Scheme 2 shows the common scavenging agents [27,32,33] and the corresponding scavenged species. For instance, p-benzoquinone was used for photo-generated electrons (e^−^) [59] and, eventually, superoxide radical (^•^O_2_^−^), sodium acetate for holes (h^+^) [60], and 2-propanol for hydroxyl radicals (^•^OH) scavenging.

**Figure 9:**
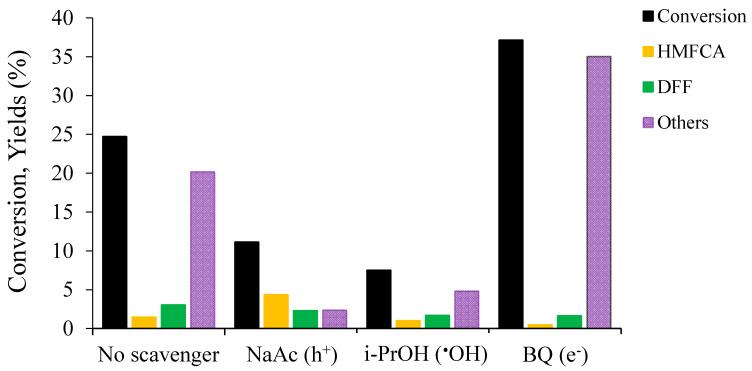
Photocatalytic performance of TiO_2_-m in presence of different scavengers. NaAc: Sodium Acetate; i-PrOH: 2-Propanol; BQ: BenzoQuinone. Reaction conditions: t = 1 h, T = 30 °C, P = 1 atm O_2_, m_cat_ = 20 mg, V = 20 mL, [HMF]_0_ = 5 × 10^−4^ mol/L, [Scavenger]/[HMF] = 9.

Figure 9 shows the photo-response of TiO_2_-m in the presence of different scavengers. The HMF conversion significantly decreased in presence of sodium acetate and 2-propanol, but the product yields were not substantially affected. This suggests that photo-generated holes and hydroxyl radicals (^•^OH) may be primary species promoting the decomposition of the substrate. On the other hand, the presence of p-benzoquinone leads to an increase in conversion, accompanied by a decrease in product yields, supporting the detrimental effect of photo-generated holes and hydroxyl radicals. This suggests that the primary agents in selective photocatalytic oxidation of HMF might be the photo-activated electrons and/or the superoxide radicals. These results are in line with previous studies of photo-catalytic HMF oxidation using g-C_3_N_4_ systems [27]. Meanwhile, the sacrificial quencher of the corresponding hole could be the oxygen of water, which is oxidized from −2 to −1, forms hydrogen peroxide and ^•^OH radicals [61].

**Figure 10:**
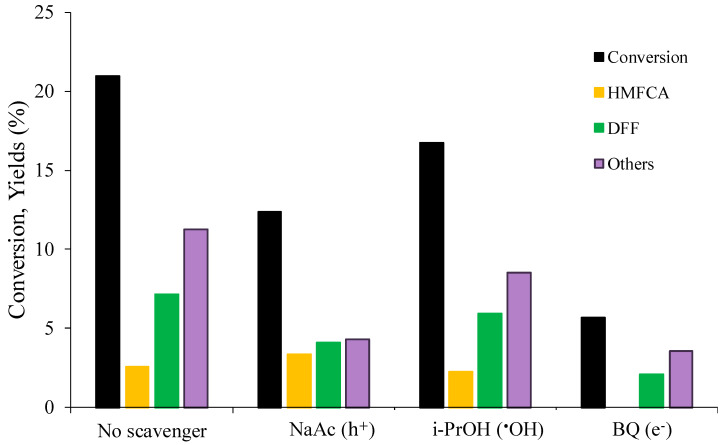
Photocatalytic performance of Au_3_Cu_1_/Ti_15_Si_85_ SFD in presence of different scavengers. NaAc: Sodium Acetate; i-PrOH: 2-Propanol; BQ: BenzoQuinone. Reaction conditions: t = 1 h, T = 30 °C, P = 1 atm O_2_, m_cat_ = 20 mg, V = 20 mL, [HMF]_0_ = 5 × 10^−4^ mol/L, [Scavenger]/[HMF] = 9.

The results of HMF photo-oxidation in presence of scavengers using of Au_3_Cu_1_/Ti_15_Si_85_ catalyst are shown in Figure 10. Similar to pristine TiO_2_-m, decorated nanocomposite revealed lower conversion of HMF in presence of sodium acetate and 2-propanol compared to the test without scavengers. However, in contrast to TiO_2_-m alone, decorated sample showed substantially lower HMF conversion in reaction with p-benzoquinone. The presence of only holes and hydroxyl radicals in the reaction did not lead to over-oxidation of HMF as in case of TiO_2_-m. This could be because the electron transfer between conduction band of titania and Au_3_Cu_1_, or Au_3_Cu_1_ and substrate, is a rate-determining step, and that electron transferred from Au_3_Cu_1_ might not participate in reduction of substrate (p-benzoquinone or oxygen), but recombine with the hole, thus generating fewer of the latter ones and hydroxyl radicals. Moreover, a lower conversion and yields of products in the reaction with p-benzoquinone suggests that also in presence of Au_3_Cu_1_ the primary species responsible for selective HMF photo-oxidation are photo-generated electrons and/or superoxide radicals.

## 3. Materials and Methods

(a) P25 (AEROXIDE TiO_2_ P25, Evonik, Essen, Germany) and DT51 (TiO_2_ CrystalACTiV DT51, TRONOX, Stamford, CT, USA) were used as commercially available references as received.
(b) Titanium(IV) butoxide (97%, Merck, Darmstadt, Germany), Triton X-100 (Alfa Aesar, Ward Hill, MA, USA), 1-hexanol (99%, Alfa Aesar, Ward Hill, MA, USA), Cyclohexane (99%, Alfa Aesar, Ward Hill, MA, USA), Ethanol (99.8%, Merck, Darmstadt, Germany), and HNO_3_ (65%, Merck, Darmstadt, Germany) were used for the synthesis of titania by reverse microemulsion method.
(c) SiO_2_ LUDOX HS-40 (40 wt%, W. R. Grace and Company, Columbia, MD, USA) for silica-titania nanocomposites prepared by spray-freeze-drying method in different ratios.
(d) HAuCl_4_ (99.995%, Merck, Darmstadt, Germany), CuSO_4_·H_2_O (99.5%, Merck, Darmstadt, Germany), Polivinilpirrolidone (PVP 25K, Merck, Darmstadt, Germany), NaOH (99%, Merck, Darmstadt, Germany), and β-D-glucose (99.9%, Merck, Darmstadt, Germany) were used for the Au_3_Cu_1_ nanoparticles preparation,
(e) 5-hydroxymethyl furfural (HMF 99%, AVA Biochem, Muttenz, Switzerland), 2,5-diformylfurane (DFF 99.99%, Toronto Reserch Chemicals, North York, ON, Canada), 5-hydroxymethyl-2-furancarboxylic acid (HMFCA 99.99%, Toronto Reserch Chemicals, North York, ON, Canada), and 5-formyl-2-furancarboxylic acid (FFCA 99.99%, Toronto Reserch Chemicals, North York, ON, Canada) were used as substrate (in case of HMF) and external standards for HPLC calibrations.
(f) Sodium acetate (99%, Merck, Darmstadt, Germany), 2-propanol (99.5%, Merck, Darmstadt, Germany), and p-benzoquinone (98%, Merck, Darmstadt, Germany) were used as radical scavengers.
(g) All of the above were used without further purification.

### 3.1. Catalyst Preparation

#### 3.1.1. Preparation of TiO_2_-m

The titanium dioxide (TiO_2_-m) was prepared via reversed microemulsion method already described elsewhere [47,62]. In brief, reversed microemulsion has been prepared adding a 5M HNO_3_ aqueous solution to a solution containing Triton X-100 (surfactant), 1-hexanol (co-surfactant), and cyclohexane (continuous oil phase) under vigorous stirring. Separately, similar amounts of Triton X-100, 1-hexanol and cyclohexane were mixed adding a titanium dioxide precursor (Titanium(IV) butoxide, TBT). The latter solution was added into a previously prepared microemulsion so that the total water-to-surfactant moles ratio was equal to 10.6. The resulting microemulsion was kept under vigorous stirring for 1 h at room temperature. The resulting microemulsion was then perturbed by heating under reflux for 5 h at 74 °C. The precipitated titania was recovered via centrifugation and washed with ethanol five times. The resulting material was either used for spray-freeze drying process in form of paste, or dried at 100 °C overnight and then calcined at 400 °C for 3 h at 2 °C/min.

#### 3.1.2. Synthesis of Au_3_Cu_1_ Nanoparticles

Metal nanoparticles were synthesized following the same procedure described in literature [53]. In summary, the necessary amount of stabilizing agent (polyvinylpyrrolidone, PVP, 2.91 × 10^−3^ mol) was added to an aqueous NaOH solution (90 mL, 5.25 × 10^−3^ mol). The solution was then heated to 95 °C in a round-bottomed flask immersed in an ethylene glycol heating bath. At this temperature, β-d-glucose and a solution (10 mL, 2.81 × 10^−3^ mol) containing the metal salts (HAuCl_4_, 3.75 × 10^−4^ mol, and CuSO_4_, 1.25 × 10^−4^ mol) were added under vigorous stirring. The synthesis of the nanoparticles was carried out for 2.5 min, then quenched immersing the flask in an ice bath to prevent the aggregation of the nanoparticles.

#### 3.1.3. Preparation of TiSi Titania-Silica Nanocomposites by Spray-Freeze-Drying (SFD)

In order to prepare composite materials, LUDOX HS-40 colloidal silica was diluted in deionized water and brought to pH = 4 using DOWEX 50X8 ion-exchange resin, filtered and added dropwise to the titania suspension under stirring. The decorated samples were prepared by adding the appropriate amount of metal nanoparticle dispersion to the titania suspension, in order to obtain a total metal loading in the resulting material of 1.5 wt%. The obtained samples were suspended in water (6 wt%) and ball milled with zirconia spheres (diameter 5 mm) for 24 h before being sprayed. The grinded suspensions were processed by using a Lab-scale Granulator LS-2 (PowderPro AB). Typically, the samples were nebulized in presence of nitrogen (0.4 bar) as auxiliary gas and sprayed by a 100-μm nozzle into a stirred solution of liquid nitrogen, thus enabling an instantaneous freezing of each generated drop. The so-frozen drops were placed into a freeze-drying equipment (LYO GT 2, SRK System Technik) with a pressure of 0.15 mbar and a temperature of −1 °C, so promoting the sublimation process which is completed within 48 h, producing a highly porous granulated powder.

### 3.2. Catalyst Characterisation

Zeta potential measurements on the colloidal samples were performed at 25 °C by electrophoretic light scattering (ELS) technique by means of the instrument Zetasizer nano ZSP (Malvern Instruments, Malvern, UK). The Smoluchowski equation was applied to convert the electrophoretic mobility to zeta potential. The instrument is equipped with an autotriation which enables the identification of the isoelectric point (IEP) and adds automatically to the sample KOH 0.1M or HCl 0.1M, in order to explore the zeta potential trend within a selected pH range. The measurments were performed on samples diluted at 0.1 wt%.

The granulated catalysts were observed by scanning electronic microscopy (SEM) using a field emission scanning electron microscope, FESEM (Carl Zeiss Sigma NTS, Oberkochen, Germany). Granules were fixed to aluminum stubs with conductive adhesive tape. Elemental analysis was performed by image analysis using FESEM coupled to an energy dispersive X-ray micro-analyzer (EDS, mod. INCA Energy 300, Oxford instruments, Abingdon, UK).

The gold-copper containing photocatalysts were also examined by high resolution transmission electron microscopy (HR-TEM), using a TEM/STEM FEI TECNAI F20 (ThermoFisher, Hillsboro, OR, USA), which uses a high-angle annular dark field (HAADF) imaging mode at 200 kV. Samples were dispersed on a holey carbon film supported on a copper grid. Image analysis was performed on more than 400 particles to calculate the particle size distribution shown as a histogram and average diameter.

XRD was measured out at room temperature with a Bragg/Brentano diffractometer (X’pertPro PANalytical, Malvern, UK) equipped with a fast X’Celerator detector, using a Cu anode as the X-ray source (Kα, λ = 1.5418 Å). Diffractograms were recorded in the range 5–80°2θ counting for 15 s every 0.05°2θ step.

Catalyst surface areas were measured by N_2_ physisorption apparatus (Sorpty 1750 CE instruments, Wigan, UK) and single point (Brunauer–Emmett–Teller) BET analysis methods, in which samples were pre-treated under vacuum at 120 °C.

Solid UV-VIS analyses were recorded in a Perkin Elmer Lambda 19 instrument (PerkinElmer, Waltham, MA, USA) equipped with integrating sphere in the range 280–800 nm to determine band gap energy (E_g_) by means of diffused reflectance spectroscopy (DRS) technique.

### 3.3. Photocatalytic Tests

The photo-oxidation of HMF has been carried out in a sealed top-irradiated glass photo-reactor (Scheme S1). Prior the reaction, 20 mL of 5 × 10^−4^ M aqueous solution of HMF with 10 mg of catalyst were stirred for 30 min in the dark, followed by oxygen purging for 5 min. The sealed reactor of 4.6 cm diameter was irradiated for 1 h under oxygen atmosphere (1 atm) using the solar simulator LOT Quantum Design LS0306 (Quantum Design, Darmstadt, Germany) that consists of 300 W Xe-lamp (irradiance of 100 mW/cm^2^ was regularly measured prior each test by radiometer HD2102.2 DELTA OHM equipped with two probes of 315–400 nm and 400–1050 nm). The temperature of the reactive solution was maintained with the circulating coolant, and kept at 30 °C during the reaction. Samples were collected at the end of the reaction, separated from photo-catalyst by centrifugation or filtration, diluted by 5 times in deionized water, and analysed with an Agilent Infinity 1200 liquid chromatograph (Agilent Technologies, Santa Clara, CA, USA) equipped with a Aminex HPX 87-H 300 mm 7.8 mm column (Bio-Rad, Hercules, CA, USA) using a 0.005 M H_2_SO_4_ solution as the mobile phase. The identification of compounds was achieved by calibration using reference commercial samples. Photocatalitic tests in presence of radical scavengers were carried out as described above, but with the addition of the radical scavenger to the HMF solution before stirring in the dark. For each scavenger, the molar ratio scavenger/HMF was kept at 9.

## 4. Conclusions

Innovative highly porous titania-silica nanocomposites with a remarkably large surface area were tested in a photocatalytic oxidation of HMF in the absence of organic solvents and base. The absence of thermal heat treatment upon the spray-freeze-drying step allowed for the deposition of pre-formed gold-copper alloy nanoparticles in an easy way, retaining their small size of around 5 nm. The photocatalytic results demonstrated that the conversion of HMF in the conditions used resulted in the formation of HMFCA and DFF among valuable products, and the overall reaction was significantly affected by the titania/silica composition and the presence of nanoparticles. The study with radical scavengers revealed that the photo-generated holes and hydroxyl radicals lead to a decomposition of both HMF and formed products, supporting the unselective character of reaction using titania in aqueous media. This tendency, however, was less significant for metal-decorated nanocomposites. On the other hand, the photo-generated electrons and/or superoxide radicals were confirmed to be primary species for the selective transformation of HMF into HMFCA and DFF for both pristine and gold-copper-containing materials.

## Figures and Tables

**Table 1:** Prepared samples and main characterization data obtained from XRD, DRS, and BET measurements. SSA_BET_: Specific Surface Area from N_2_ physisorption; *ρ*: powder bulk density; d: average crystallite size with respect to anatase (101) reflection; E_g_: band gap energy from DRS. Metal total loading of Au_3_Cu_1_ is 1.5 wt%.

Catalyst	Crystalline Phase	SSA_BET_ (m^2^/g)	*ρ* (g/cm^3^)	d (nm)	E_g_ (eV)
P25	80% Anatase 20% Rutile	49	0.10	23	3.10
DT51	Anatase	80	0.36	20	3.26
TiO_2_-m	Anatase	130	1.30	8	3.08
TiO_2_-m SFD		230	0.10	3	3.04
Ti_50_Si_50_ SFD		250	0.05	3	2.92
Ti_25_Si_75_ SFD		240	0.04	3	3.48
Ti_15_Si_85_ SFD		210	0.03	2	3.56
Au_3_Cu_1_/Ti_15_Si_85_ SFD		140	0.04	5.0 *	3.56
Au_3_Cu_1_/TiO_2_-m SFD		180	0.09	4.6 *	3.04

* Average dimension of Au_3_Cu_1_ nanoparticles determined by HAADF-STEM.

**Table 2:** Photocatalytic results of tested catalysts. Reaction conditions: t = 1 h, T = 30 °C, P = 1 atm O_2_, m_cat_ = 20 mg, V = 20 mL, [HMF]_0_ = 5 × 10^−4^ mol/L.

Catalyst	Conversion HMF (%)	Yield HMFCA (%)	Selectivity HMFCA (%)	Yield DFF (%)	Selectivity DFF (%)	Others (%)
P25	94	1	1	1	1	92
DT51	62	1	1	4	6	57
TiO_2_-m	25	1	4	3	13	21
TiO_2_-m SFD	12	1	7	4	34	7
Ti_50_Si_50_ SFD	7	1	2	2	42	4
Ti_25_Si_75_ SFD	11	1	3	3	25	7
Ti_15_Si_85_ SFD	24	1	2	3	13	20
Au_3_Cu_1_/TiO_2_-m SFD	3	0	0	2	67	1
Au_3_Cu_1_/Ti_15_Si_85_ SFD	21	3	12	7	34	11

## Supplementary Materials

The following are available online. Scheme S1: Solar simulator set-up, Figure S1: SEM images of different TiO_2_ powders, Figure S2: HAADF-STEM analysis and EDX mapping of Au_3_Cu_1_/TiO_2_-m SFD, Figure S3: Normalized HMF conversion rates and HMFCA and DFF production rates, Figure S4: Photocatalytic activity of microemulsion titania decorated via incipient wetness impregnation with different metal nanoparticles.
