# Supplementary material for: Photocatalytic Oxidation of HMF under Solar Irradiation: Coupling of Microemulsion and Lyophilization to Obtain Innovative TiO2-Based Materials
Source: Molecules. 2020 Nov 10;25(22):5225. doi: 10.3390/molecules25225225 (PMC7696902; doi:10.3390/molecules25225225)
Supplement: Supplementary file 1 [file molecules-25-05225-s001.pdf]

molecules

Supplementary Materials

Photocatalytic Oxidation of HMF under solar irradiation: coupling of microemulsion and lyophilization to obtain innovative TiO_2-based materials

Alessandro Allegri [1], Valeriia Maslova [1,*], Magda Blosi [2], Anna Luisa Costa [2], Simona Ortelli [2], Francesco Basile [1] and Stefania Albonetti [1,2,*]

[1] Dip. Chimica Industriale "Toso Montanari", Università di Bologna, Viale Risorgimento 4, 40136 Bologna (BO), Italy; alessandro.allegri2@unibo.it (A.A.); f.basile@unibo.it (F.B.)
[2] ISTEC-CNR, Institute of Science and Technology for Ceramics, National Research Council, Via Granarolo 64, 48018 Faenza, Italy; magda.blosi@istec.cnr.it (M.B.); anna.costa@istec.cnr.it (A.L.C.); simona.ortelli@istec.cnr.it (S.O.)
* Correspondence: valeriia.maslova2@unibo.it (V.M.); stefania.albonetti@unibo.it (S.A.)

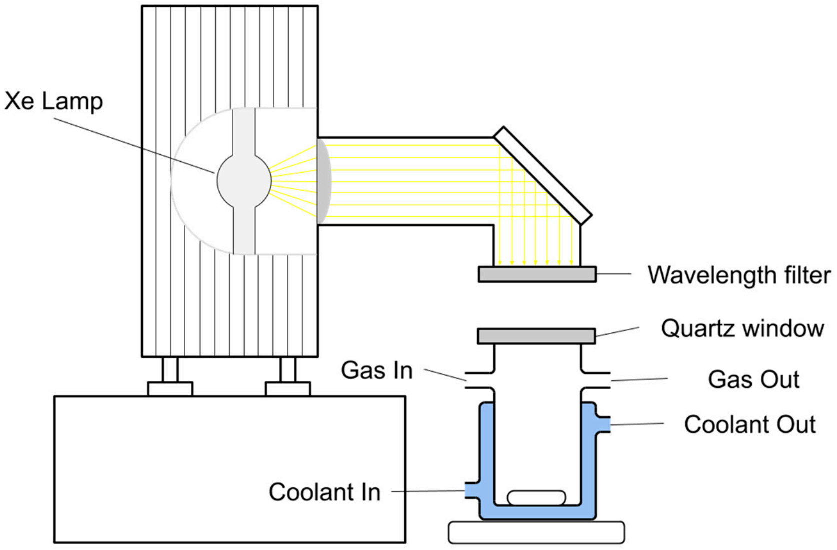

Scheme S1. Solar simulator set-up.

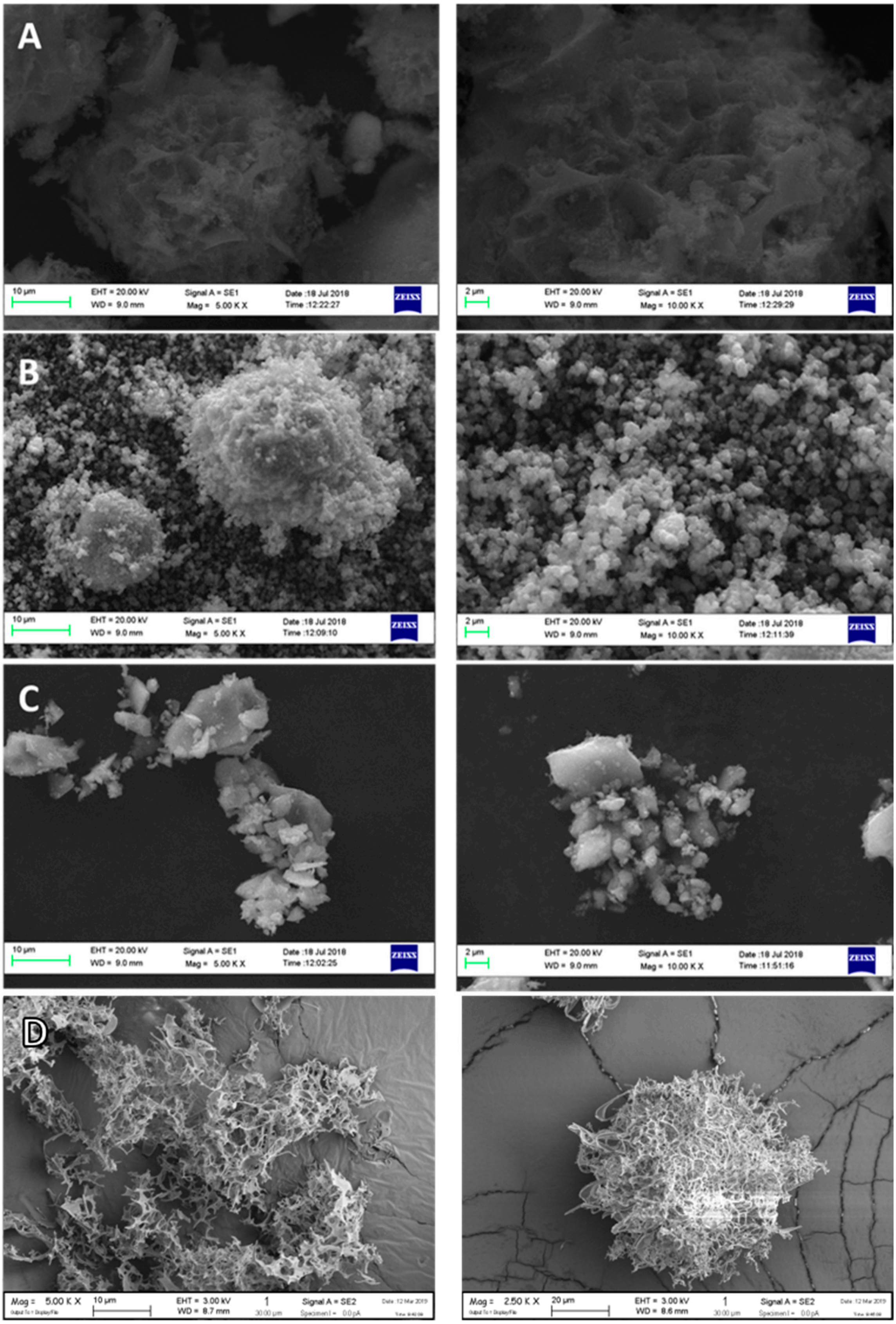

Figure 1. SEM images of different TiO_2 powders. **A**–P25, **B**–DT51, **C**–TiO_2-m, **D**–TiO_2-m SFD.

SEM images of P25, DT51 and TiO_2-m showed differences in microstructure. The commercial DT51 has homogenously distributed small, round-shape particles, while the particles of P25 has a

very porous, sponge-like form. The synthesized TiO_2-m consists of small, but highly aggregated particles. Using SFD process for TiO_2-m SFD production, avoid powder sintering, leading to material with high surface area.

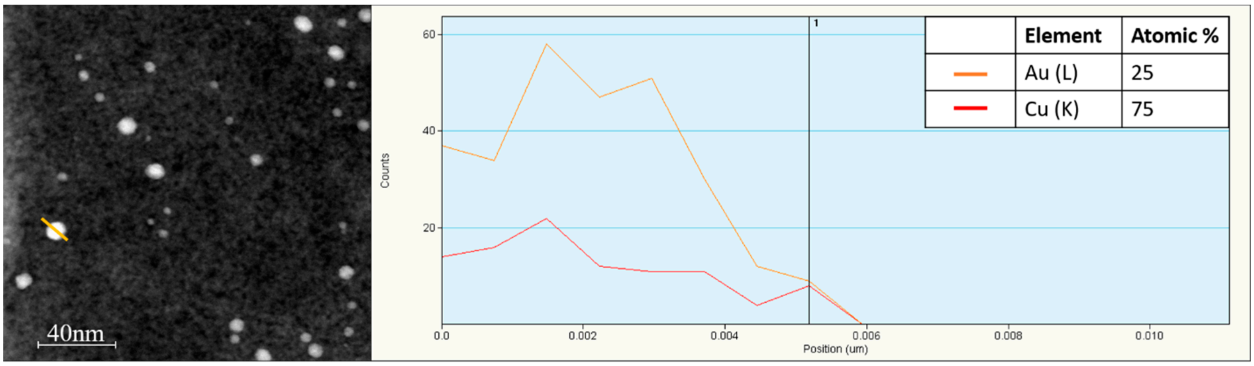

Figure 2. HAADF-STEM analysis and EDX mapping of Au_3Cu_1/TiO_2-m SFD.

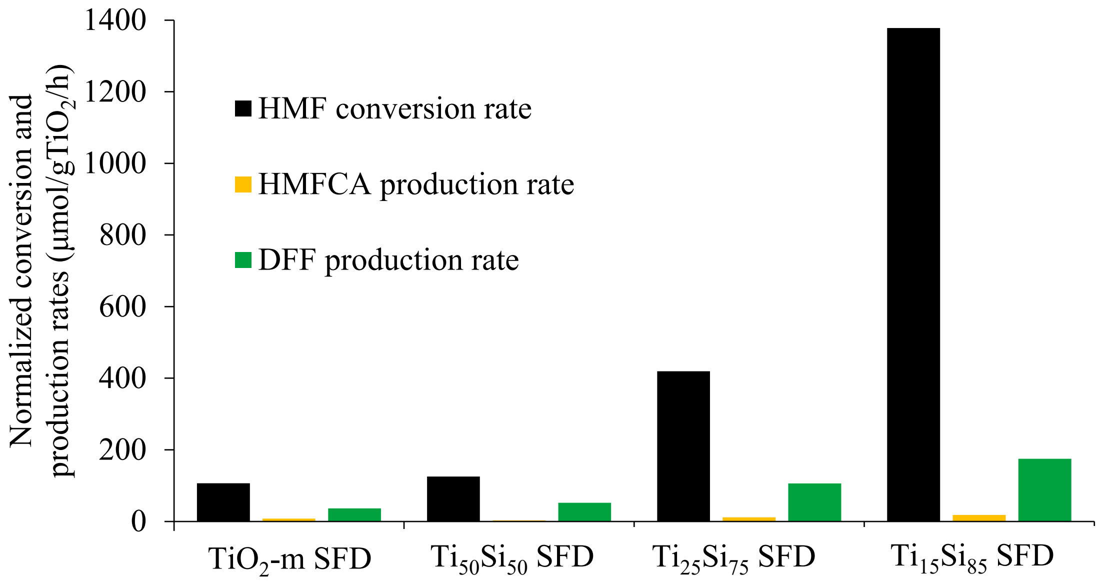

Figure 3. Normalized HMF conversion rates and HMFCA and DFF production rates.

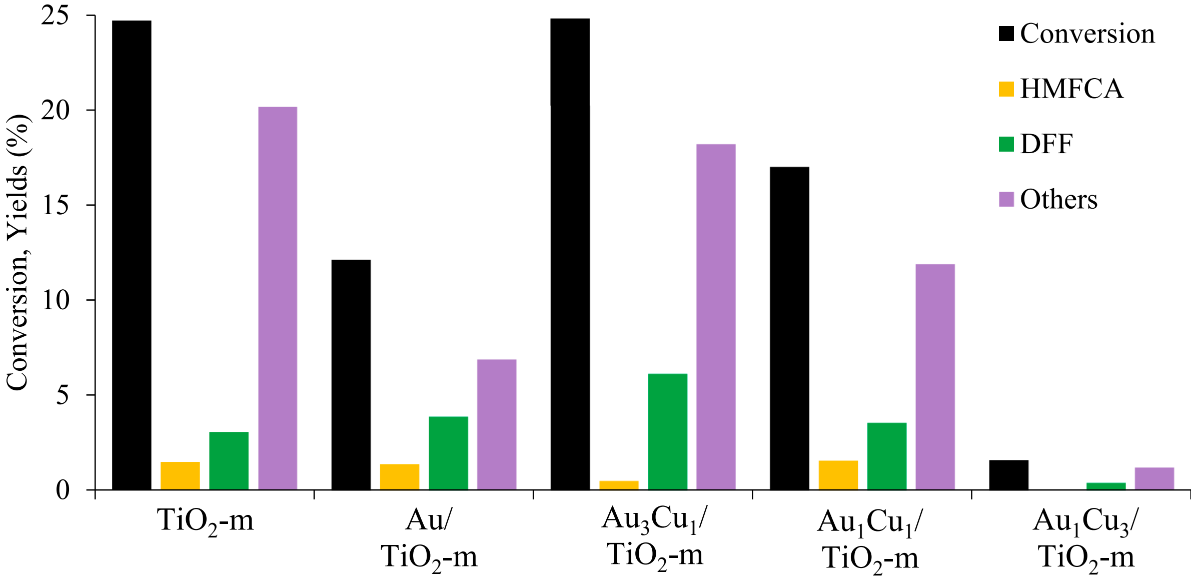

Figure 4. Photocatalytic activity of microemulsion titania decorated via incipient wetness impregnation with different metal nanoparticles. Reaction conditions: t = 1h, T=30 °C, P=1atm O_2, m_{cat} = 20 mg, V = 20mL, $[HMF]_0$=5 × 10^{-4}mol/L.
